# Identification of HLA-A2-restricted CTL epitopes of a novel tumour-associated antigen, KIF20A, overexpressed in pancreatic cancer

**DOI:** 10.1038/sj.bjc.6606052

**Published:** 2010-12-21

**Authors:** K Imai, S Hirata, A Irie, S Senju, Y Ikuta, K Yokomine, M Harao, M Inoue, Y Tomita, T Tsunoda, H Nakagawa, Y Nakamura, H Baba, Y Nishimura

**Affiliations:** 1 Department of Immunogenetics, Graduate School of Medical Sciences, Kumamoto University, Kumamoto, Japan; 2 Department of Gastroenterological Surgery, Graduate School of Medical Sciences, Kumamoto University, Kumamoto, Japan; 3 Laboratory of Molecular Medicine, Human Genome Center, Institute of Medical Science, The University of Tokyo, Tokyo, Japan

**Keywords:** anticancer immunotherapy, tumour-associated antigen, CTL, KIF20A/RAB6KIFL/MKlp2, HLA-transgenic mouse

## Abstract

**Background:**

Identification of tumour-associated antigens (TAAs) that induce cytotoxic T lymphocytes (CTLs) specific to cancer cells is critical for the development of anticancer immunotherapy. In this study, we aimed at identifying a novel TAA of pancreatic cancer for immunotherapy.

**Methods:**

On the basis of the genome-wide cDNA microarray analysis, we focused on KIF20A (also known as RAB6KIFL/MKlp2) as a candidate TAA in pancreatic cancer cells. The HLA-A2 (A*02:01)-restricted CTL epitopes of KIF20A were identified using HLA-A2 transgenic mice (Tgm) and the peptides were examined to check whether they could generate human CTLs exhibiting cytotoxic responses against KIF20A^+^, HLA-A2^+^ tumour cells *in vitro*.

**Results:**

*KIF20A* was overexpressed in pancreatic cancer and in some other malignancies, but not in their non-cancerous counterparts and many normal adult tissues. We found that KIF20A-2 (p12–20, LLSDDDVVV), KIF20A-8 (p809–817, CIAEQYHTV), and KIF20A-28 (p284–293, AQPDTAPLPV) peptides could induce HLA-A2-restricted CTLs in HLA-A2 Tgm without causing autoimmunity. Peptide-reactive human CTLs were generated from peripheral blood mononuclear cells of HLA-A2^+^ healthy donors by *in vitro* stimulation with the three peptides, and those CTLs successfully exhibited cytotoxic responses to cancer cells expressing both KIF20A and HLA-A2.

**Conclusion:**

KIF20A is a novel promising candidate for anticancer immunotherapeutic target for pancreatic cancers.

Pancreatic cancer is one of the highly lethal malignancies with an overall 5-year survival rate of ∼5% (Jemal *et al*, 2007). Although a surgical resection is the only treatment for long-term survival, patients with resectable pancreatic cancer are in the minority (9–22%) (Eloubeidi *et al*, 2006; Goonetilleke and Siriwardena, 2007). Furthermore, even the 5-year survival rate after a curative resection is reported to be ∼20% (Cleary *et al*, 2004; Smeenk *et al*, 2005; Moon *et al*, 2006). Therefore, there is a strong need for development of novel therapeutic modalities.

Anticancer immunotherapy is considered to be the candidate modality for pancreatic cancer. Recently, analyses of gene expression profiles of cancer and normal cells using cDNA microarray technologies have provided an effective approach for the identification of tumour-associated antigens (TAAs) (Nakatsura *et al*, 2004b; Uchida *et al*, 2004; Yoshitake *et al*, 2004; Watanabe *et al*, 2005; Komori *et al*, 2006; Suda *et al*, 2006; Harao *et al*, 2008; Imai *et al*, 2008). This study analysed the gene expression profiles of pancreatic cancer using a genome-wide cDNA microarray consisting of 27 648 genes, which revealed that *KIF20A* was overexpressed in pancreatic cancer tissues but not in many normal tissues.

In this study, we examined whether KIF20A could be a potential target for anticancer immunotherapy. To this aim, human KIF20A-derived and HLA-A2-restricted cytotoxic T lymphocyte (CTL) epitopes were identified using HLA-A2 transgenic mice (Tgm), and the ability of peptides to induce KIF20A-reactive human CTLs that kill cancer cells and the safety not to induce autoimmune responses in the mouse were investigated.

## Materials and methods

### cDNA microarray analysis

A data set of genome-wide cDNA microarray analyses using cancerous and adjacent normal tissues obtained by a laser microbeam dissection (Nakamura *et al*, 2004) was used in this study. The tissue samples were obtained from surgical specimens of pancreatic cancer patients. All patients provided their written informed consent to participate in this study.

### Mice, cell lines, and HLA expression

The HLA-A2 Tgm, H-2D^b−/−^ *β*_2_m^−/−^ double knockout mice introduced with a monochain gene construct of human *β*2m-HLA-A2.1(*α*1, *α*2)-H-2D^b^(*α*3, transmembrane, and cytoplasmic) (Pascolo *et al*, 1997; Firat *et al*, 1999), were kindly provided by Dr FA Lemonnier. Mice were maintained and handled in accordance with the animal care guidelines of the Kumamoto University.

The human pancreatic cancer cell line PANC1, the colon cancer cell line CaCo-2, and a transporter associated with antigen processing (TAP)-deficient and HLA-A2 (A*02:01)-positive cell line T2 were purchased from the Riken Cell Bank (Tsukuba, Japan). The human liver cancer cell line SKHep1 and the human pancreatic cancer cell line PK9 were provided by Dr Kyogo Itoh (Kurume University, Kurume, Japan) and the Cell Resource Center for Biomedical Research Institute of Development, Aging and Cancer (Tohoku University, Sendai, Japan), respectively.

The expression of HLA-A2 was examined by flow cytometry with an anti-HLA-A2 monoclonal antibody (mAb), BB7.2 (One Lambda Inc., Canoga Park, CA, USA) to select HLA-A2-positive blood donors and target cancer cell lines.

### Patients, blood samples, and tumour tissues

The clinical research using peripheral blood mononuclear cells (PBMCs) obtained from healthy donors was approved by the Institutional Review Board of the Kumamoto University. The cancer and adjacent non-cancerous tissues were obtained from 14 patients during routine diagnostic procedures from patients in the Kumamoto University Hospital. The tissues were subjected to either reverse transcription–PCR (RT–PCR) or immunohistochemical analyses as listed in Table 1. Blood and tissue samples were obtained from donors and patients, respectively, after receiving their written informed consent.

### RT–PCR

Reverse transcription–PCR analyses were performed as described previously (Nakatsura *et al*, 2004a). The primers used were: *KIF20A*, sense 5′-CTACAAGCACCCAAGGACTCT-3′ (788-808, the 4th exon) and antisense 5′-AGATGGAGAAGCGAATGTTT-3′ (1400-1381, the 8th exon) and *ACTB*, sense 5′-CATCCACGAAACTACCTTCAACT-3′ (903-925, the 5th exon) and antisense 5′-TCTCCTTAGAGAGAAGTGGGGTG-3′ (1535-1513, the 6th exon). Primer positions are shown according to cDNA sequences presented in the Gene Bank Accession numbers of NM_005733 for human *KIF20A* and of NM_001101 for human *ACTB*. The products were 612 bp long for *KIF20A* and 632 bp long for *ACTB*. After normalisation by the intensity for *ACTB* mRNA, the expression levels of *KIF20A* mRNA were compared among the tissues and cell lines.

### Western blot analysis and immunohistochemical examination

Western blot analysis and immunohistochemical staining of KIF20A using rabbit polyclonal anti-KIF20A antibody (category no. A300-879A) of Bethyl Laboratories (Montgomery, TX, USA) were performed as described previously (Nakatsura *et al*, 2001; Yoshitake *et al*, 2004). Immunohistochemical staining of CD4 or CD8 in tissue specimens of HLA-A2 Tgm immunised with the KIF20A-8_809–817_ peptide was performed as described previously (Matsuyoshi *et al*, 2004).

### Lentiviral gene transfer

*KIF20A* cDNA was transduced into SKHep1 cells by a lentiviral vector-mediated gene transfer as described previously (Tahara-Hanaoka *et al*, 2002; Irie *et al*, 2006). The expression of KIF20A was confirmed by western blot analysis.

### Induction and response of KIF20A-reactive mouse CTLs

A total of 36 human KIF20A-derived peptides (purity >95%) with predicted high binding scores for HLA-A2 (A*02:01) by BIMAS software (NIH, Bethesda, MD, USA; http://www-bimas.cit.nih.gov/) were synthesised by the American Peptide Company (Sunnyvale, CA, USA; Supplementary Table 1). The HLA-A2 Tgm were immunised intraperitoneally twice on days 0 and 7 with bone marrow-derived dendritic cells (BM-DCs) pulsed with 12 sets of a mixture of 3 kinds of the 36 synthesised peptides. Seven days after the last immunisation, CD4-depleted spleen cells (CD4^−^ spleen cells) from Tgm using CD4 microbeads (Miltenyi Biotec, Auburn, CA, USA) were stimulated *in vitro* with BM-DCs pulsed with each peptide. The CTL responses to the peptides were tested by the ELISPOT assay (human INF-*γ* ELISPOT kit, BD Biosciences, Franklin Lakes, NJ, USA).

### Induction of KIF20A-reactive human CTLs

Peripheral monocyte-derived DCs were generated from CD14^+^ cells isolated from PBMCs of HLA-A2-positive healthy donors using CD14 microbeads (Miltenyi Biotec), with stimulation of 100 ng ml^−1^ granulocyte/macrophage colony-stimulating factor, 10 ng ml^−1^ interleukin (IL)-4, and Streptococcal OK-432 (Picibanil, Chugai Pharmaceutical Co., Ltd., Tokyo, Japan) (Naito *et al*, 2006) as described previously (Harao *et al*, 2008). The cells were morphologically changed to express many dendrites, and their expression levels of MHC class-II and CD80 were upregulated after the stimulation with OK-432. The DCs were pulsed with 20 *μ*g ml^−1^ candidate peptides in the presence of 4 *μ*g ml^−1^ *β*2-microglobulin (Sigma-Aldrich, St Louis, MO, USA) for 2 h at 37°C in AIM-V medium (Invitrogen, Carlsbad, CA, USA) containing 2% autologous plasma. The DCs were then irradiated (40 Gy) and incubated with CD8^+^ cells isolated from the same PBMCs using CD8 microbeads (Miltenyi Biotec). These cultures were set up in 24-well plates; each well contained 1 × 10^5^ peptide-pulsed DCs, 2 × 10^6^ CD8^+^ T cells, and 5 ng ml^−1^ human IL-7 (Wako, Osaka, Japan), in 2 ml AIM-V with 2% autologous plasma. After 2 days, these cultures were supplemented with human IL-2 (PeproTec, Rocky Hill, NJ, USA) to a final concentration of 20 IU ml^−1^. Two additional stimulations with peptide-loaded autologous DCs were carried out on days 7 and 14. Six days after the last stimulation, antigen-specific CTL responses were investigated by the ELISPOT assay and the ^51^Cr release assay as described previously (Komori *et al*, 2006).

### CTL responses against cancer cell lines

Human CTLs were co-cultured with cancer cells or peptide-pulsed T2 cells as a target (5 × 10^3^ per well) at the indicated effector/target ratio, and a standard ^51^Cr release assay was performed (Komori *et al*, 2006). An HLA-A2 (A*02:01)-binding HIV-derived peptide, SLYNTVATL, was used as an irrelevant control peptide (Parker *et al*, 1992). Blocking of HLA-class I or HLA-DR in the ELISPOT assay was performed as follows: the target cancer cells were incubated with 10 *μ*g ml^−1^ anti-HLA-class I mAb, W6/32, or 10 *μ*g ml^−1^ anti-HLA-DR mAb, H-DR-1 (kindly provided by Dr Kyogo Itoh (Nakao *et al*, 1995)) for 1 h before co-culture with CTLs, and the inhibitory effects of mAbs on the production of IFN-*γ* by CTLs were monitored (Gomi *et al*, 1999).

## Results

### Identification of the *KIF20A* gene upregulated in pancreatic cancer and various malignancies based on cDNA microarray analyses

**Figure 1:**
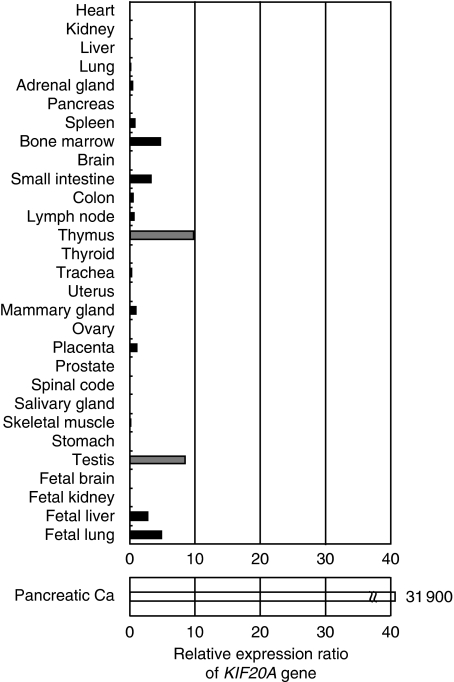
The relative expression ratio of *KIF20A* mRNA in pancreatic cancer tissues (Pancreatic Ca) and in various normal tissues based on a cDNA microarray analysis. The *KIF20A* gene was overexpressed in all six pancreatic cancer tissues investigated, but barely expressed in many normal tissues, except for in the testis and thymus. The ratio of relative expression levels of *KIF20A* mRNA in six pancreatic cancer tissues to that of disease-free counterparts was calculated. For normal tissues, the average expression level of *KIF20A* mRNA in all tissues was assigned to be 1.0, and the relative expression level of *KIF20A* mRNA in each tissue was calculated. For pancreatic cancer, the expression levels of *KIF20A* mRNA in adjacent normal pancreatic tissues were assigned to be 1.0, and the relative expression levels of *KIF20A* mRNA in pancreatic cancers were calculated using specimens obtained from six patients with pancreatic cancer.

Using genome-wide cDNA microarray analyses, it turned out that six genes, namely *CDH3*, *KIF20A*, *MICAL2*, *TRIM29*, *ABHD*, and *EPHA4*, were overexpressed in the six pancreatic cancer tissues in comparison with their adjacent normal counterparts, and we reported that CDH3/P-cadherin is a new TAA for immunotherapy of pancreatic, gastric, and colorectal cancers (Imai *et al*, 2008). In this study, we focused on *KIF20A* as another pancreatic cancer-specific TAA for immunotherapeutic target. The cDNA microarray analyses revealed that expression of the *KIF20A* gene in pancreatic cancer tissues was markedly enhanced in all six patients investigated (the average of the relative expression ratio: 31 900, ranging 15–72 000), whereas the *KIF20A* gene was faintly expressed only in the testis and thymus among normal tissues (Figure 1). In addition, overexpression of the *KIF20A* gene was also observed in other malignancies, such as lung and bladder cancers (Table 2) (Kitahara *et al*, 2001; Hasegawa *et al*, 2002; Kikuchi *et al*, 2003; Nakamura *et al*, 2004; Obama *et al*, 2005) .

### Expression of *KIF20A* mRNA and protein in normal organs, cancer cell lines, and pancreatic cancer tissues

**Figure 2:**
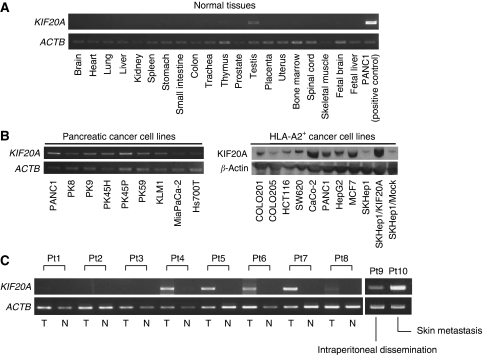
Expression of *KIF20A* mRNA and protein in human normal tissues, cancer cell lines, and pancreatic cancer tissues. (**A**) RT–PCR analysis of *KIF20A* mRNA expression in various normal tissues. *KIF20A* mRNA was not detected except for faint expression observed in the testis. (**B**) The expression of *KIF20A* mRNA and protein in various cancer cell lines investigated by RT–PCR (left) and western blot analyses (right). *KIF20A* mRNA was detected in all pancreatic cancer cell lines examined. The major bands at ∼100 kDa, which corresponds to the calculated molecular weight for KIF20A, are shown. It must be noted that SKHep1 and SKHep1/Mock expressed only a trace amount of the KIF20A protein, whereas *KIF20A* cDNA-transfected SKHep1, SKHep1/KIF20A, relatively expressed a large amount of the protein. (**C**) RT–PCR analyses of the *KIF20A* expression in pancreatic cancer tissues (T), and their normal counterparts (N). The expression of the *KIF20A* mRNA was detected in five of eight pancreatic tumour tissues, but little expression was detected in their normal counterparts. It must be noted that *KIF20A* mRNA expression was also detected in the peritoneum (patient 9 (Pt9)) and metastatic foci of the skin (patient 10 (Pt10)).

The expression of the *KIF20A* gene in normal tissues was analysed by RT–PCR analyses, which revealed its exclusive expression in the testis and thymus (Figure 2A). On the other hand, the expression of the *KIF20A* gene was detected in almost all pancreatic and other HLA-A2-positive cancer cell lines tested (Figure 2B, left). Those observations essentially coincided with data obtained by cDNA microarray analyses.

We then checked the expression of the *KIF20A* gene in surgically resected pancreatic cancer tissues and their adjacent normal counterparts by RT–PCR analyses. The *KIF20A* gene was detected in five of eight pancreatic cancer tissues, whereas virtually no expression was observed in their normal counterparts (Figure 2C). It is noteworthy that *KIF20A* was also detected in the metastatic foci of the skin and peritoneum.

**Figure 3:**
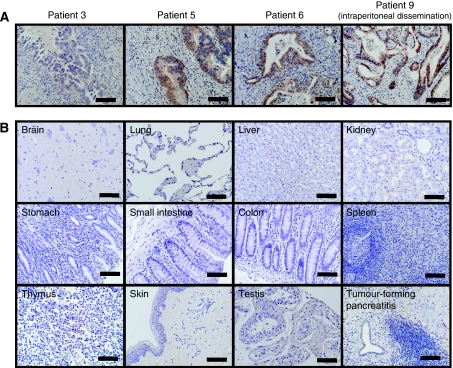
Immunohistochemical analyses of the KIF20A protein in (**A**) pancreatic cancer and in (**B**) various normal tissues. (Panel **A**) A strong staining of KIF20A was mainly observed at the cytoplasm and nuclei of cancer cells in six of nine cases (representative patients 5, 6, and 9 are shown), whereas a very weak staining was observed in acinar cells and in the normal ductal epithelium of their normal adjacent pancreatic tissues. A pancreatic cancer tissue without KIF20A expression is also shown (Patient 3). A similar strong staining was observed in the metastatic foci of the peritoneum (Patient 9). (Panel **B**) KIF20A was not detected in the normal brain, lung, liver, kidney, stomach, small intestine, colon, spleen, skeletal muscle, skin, thymus, and testis. Little staining was detected in tumour-forming pancreatitis. Positive staining signals are seen as brown. The scale bars represent 100 *μ*m.

Western blot analysis revealed expression of the KIF20A protein in various HLA-A2^+^ cancer cell lines tested, except for SKHep1 (Figure 2B, right). To confirm the tumour-associated overexpression of the KIF20A protein, various paraffin-embedded normal tissue specimens and pancreatic cancer specimens were examined by immunohistochemical analyses. We investigated nine samples of pancreatic cancer (Table 1), and a strong staining of KIF20A was mainly observed in the cytoplasm and nuclei of cancer cells in six cases, whereas a very weak staining was observed in acinar cells and the normal ductal epithelium of their normal adjacent pancreatic tissues (Figure 3A). Little staining was detected in tumour-forming pancreatitis. KIF20A was not detected in the normal brain, lung, liver, kidney, stomach, small intestine, colon, spleen, skeletal muscle, skin, thymus, and skin (Figure 3B). Therefore, the pattern of KIF20A protein expression essentially paralleled with its gene expression profile.

### Identification of KIF20A-derived and HLA-A2-restricted mouse CTL epitopes using HLA-A2 Tgm

**Figure 4:**
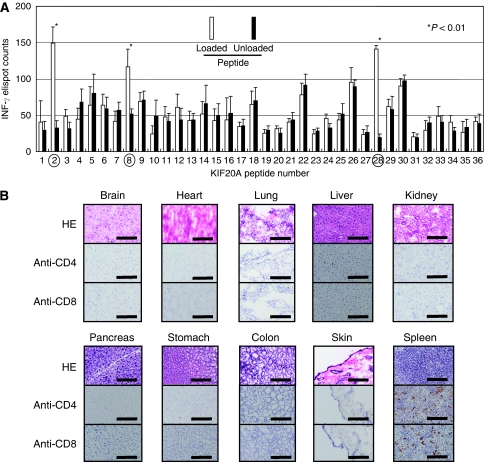
Identification of HLA-A2-restricted mouse CTL epitopes of human KIF20A using HLA-A2 Tgm. (**A**) HLA-A2 Tgm were immunised with syngeneic BM-DCs pulsed with the peptide mixtures as described in the ‘Materials and Methods’ section, and CD4^−^ spleen cells were stimulated with BM-DCs pulsed with or without each peptide for 6 days. INF-*γ*-producing CTLs were detected by an ELISPOT assay. KIF20A-2 (LLSDDDVVV), KIF20A-8 (CIAEQYHTV), and KIF20A-28 (AQPDTAPLPV) peptides indicated by the circles were shown to induce peptide-reactive CTLs. These assays were performed twice with similar results. (**B**) Immunohistochemical staining with anti-CD4 or anti-CD8 mAb in tissue specimens of HLA-A2 Tgm immunised with the KIF20A-8 peptide. After two-times vaccinations, these specimens were removed and analysed.

A total of 36 peptides with high binding scores to HLA-A2 (A*02:01) by BIMAS (Supplementary Table 1) were immunised to HLA-A2 Tgm, and CD4^−^ spleen cells were stimulated *in vitro* with BM-DCs pulsed with each peptide. The CD4^−^ spleen cells stimulated with KIF20A-2 (p12–20, LLSDDDVVV), KIF20A-8 (p809–817, CIAEQYHTV), and KIF20A-28 (p284–293, AQPDTAPLPV) peptides produced a significant amount of INF-*γ* in a peptide-specific manner in the ELISPOT assay (Figure 4A). Those CD4^−^ spleen cells (2 × 10^4^) showed 149.0±22.2, 117.2±23.4, and 141.2±5.5 spot counts per well in response to BM-DCs pulsed with the KIF20A-2, KIF20A-8, and KIF20A-28 peptides, respectively, whereas they showed 32.6±9.9, 51.4±7.8, and 19.2±5.2 spot counts per well, respectively, without peptide loading (*P*<0.01). No significant peptide-specific response was observed with the other peptides. These results suggest that the KIF20A-2, KIF20A-8, and KIF20A-28 peptides could be the HLA-A2-restricted CTL epitope peptides in HLA-A2 Tgm and those peptides were expected to be epitopes for human CTLs.

### No autoimmune phenomenon induced by immunisation with KIF20A-8 in HLA-A2 Tgm

Whether immunisation with KIF20A peptides induces autoimmune reactions is of great importance. The immunohistochemical analyses of several vital organs with anti-CD4 and anti-CD8 mAbs were performed in HLA-A2 Tgm after two-times vaccination with the KIF20A-8 peptide, as its amino-acid sequence was identical between human and mouse KIF20A. As shown in Figure 4B, no pathological change that suggests autoimmunity, such as lymphocyte infiltration or tissue destruction, was observed, indicating that lymphocytes stimulated with the KIF20A-8 peptide were safe at least in HLA-A2 Tgm.

### Induction of KIF20A-reactive human CTLs from PBMCs of HLA-A2-positive healthy donors

**Figure 5:**
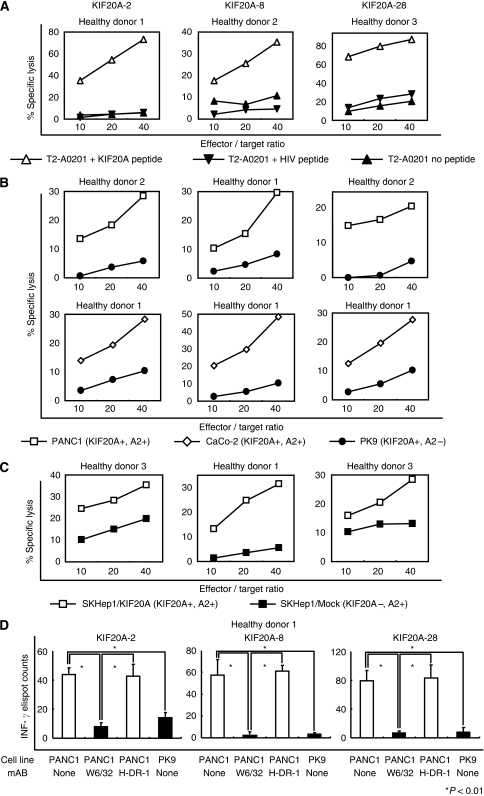
Induction of KIF20A-specific human CTLs from PBMCs of three HLA-A2-positive healthy donors. (**A**) KIF20A peptide-reactive CTLs generated from PBMCs of three HLA-A2-positive healthy donors effectively killed T2 cells (HLA-A2^+^, TAP deficient) pulsed with each peptide but not those unpulsed or pulsed with irrelevant and HLA-A2-restricted CTL epitopes of HIV. (**B**) Human CTLs exhibited cytotoxicity to the KIF20A^+^, HLA-A2^+^ human pancreatic cancer cell line PANC1 and to the colon cancer cell line CaCo-2, but not to KIF20A^+^, HLA-A2^−^ human pancreatic cancer cell line PK9. (**C**) The cytotoxicity of human CTLs was KIF20A specific. Those CTLs killed SKHep1/KIF20A, but not mock-transfected SKHep1 cells. Representative data are shown. (**D**) Human CTL responses were inhibited by anti-HLA-class I mAb (W6/32, IgG_2a_) but not by anti-HLA-DR mAb (H-DR-1, IgG_2a_). The target cells used were PANC-1cell (KIF20A^+^, HLA-A2^+^) and PK9 cell (KIF20A^+^, HLA-A2^−^). (Panels **A**–**D**) Representative data from one of the three donors with similar results are shown.

To investigate whether the KIF20A-2, KIF20A-8, and KIF20A-28 peptides could generate KIF20A-specific human CTLs, CD8^+^ T cells sorted from PBMCs of HLA-A2-positive healthy donors were incubated with the autologous CD14^+^ cell-derived DCs pulsed with each peptide. After three-times stimulations, the cytotoxic activities of CD8^+^ T cells against peptide-pulsed T2 cells were examined by the ^51^Cr release assay (Figure 5A). Each CTL killed the T2 cells pulsed with KIF20A-2 (left), KIF20A-8 (middle), or KIF20A-28 (right) peptides, but not the T2 cells pulsed with irrelevant HLA-A2-binding HIV peptide or those without peptide loading. The data indicate that KIF20A peptides successfully induced human CTLs with peptide-specific cytotoxicity.

Next, the capacity of these CTLs to kill human cancer cell lines expressing both KIF20A and HLA-A2 was examined. As shown in Figure 5B, KIF20A-reactive CTLs stimulated with KIF20A-2 (left), KIF20A-8 (middle), or KIF20A-28 (right) peptides exhibited effective cytotoxicity to PANC1 (KIF20A^+^, HLA-A2^+^) and CaCo-2 (KIF20A^+^, HLA-A2^+^), but not to PK9 (KIF20A^+^, HLA-A2^−^). Furthermore, all CTLs exhibited cytotoxicity against SKHep1/KIF20A (KIF20A^+^, HLA-A2^+^), SKHep1 cells (KIF20A^−^, HLA-A2^+^) transfected with the *KIF20A* gene (Figure 2B, right), but not against SKHep1/Mock, SKHep1 cells transfected with an empty vector (Figure 5C). Those results suggested that CTL responses were specific to KIF20A expression and that the epitope peptides were naturally processed and expressed on the surface of cancer cells in the context of HLA-A2 molecules.

Blocking mAb specific to HLA-class I (W6/32) markedly reduced the number of INF-*γ*-producing CTLs generated by stimulation with KIF20A-2 (left), KIF20A-8 (middle), or KIF20A-28 (right) peptides by co-culture with PANC1 cells with statistical significance (Figure 5D, *P*<0.01), whereas anti-HLA-DR mAb (H-DR-1) had no effect on CTL responses. The data verified that CTLs recognised KIF20A-expressing target cells in an HLA-class I-restricted manner.

## Discussion

In this study, whether KIF20A is applicable to a target of anticancer immunotherapy was carefully investigated. The *KIF20A* gene was overexpressed in pancreatic cancer cells but barely expressed in their normal counterparts and in many normal adult tissues as revealed by the cDNA microarray analysis, RT–PCR analysis, and immunohistochemical analyses. As a weak expression of *KIF20A* in the normal thymus in addition to the testis was observed in the cDNA microarray and RT–PCR analyses, KIF20A is not a perfect cancer/testis antigen but a TAA overexpressed in pancreatic cancer, as well as in bladder cancer, non-small cell lung cancer, and cholangiocellular carcinoma. We have reported that CDH3/P-cadherin is another TAA that is overexpressed in pancreatic cancer (Imai *et al*, 2008); however, it has been often observed that using a single TAA as a target of anticancer immunotherapy does not yield satisfactory therapeutic outcomes in animal models. Using multiple TAAs in combination as targets drastically improved the outcome of anticancer immunotherapy (Fukushima *et al*, 2009), and therefore KIF20A could be not only a versatile tumour marker but also a TAA useful as a target of anticancer immunotherapy for pancreatic cancer by itself or in combination with other TAA(s). Furthermore, the involvement of KIF20A in pancreatic carcinogenesis suggests that KIF20A would be a promising immunotherapeutic target for pancreatic cancer as described below.

The KIF20A protein, also known as RAB6KIFL/MKlp2, was first identified to localise to Golgi apparatus and to have an important role in the dynamics in this organelle by an interaction with the GTP-bound form of Rab6 (Echard *et al*, 1998). KIF20A belongs to a large family of motor proteins that accumulate in mitotic cells, and microinjection of anti-KIF20A antibodies resulted in the failure of cytokinesis (Hill *et al*, 2000). The knockdown of *KIF20A* gene expression in pancreatic cancer cell lines by small-interfering RNA drastically inhibited the growth of those cells (Taniuchi *et al*, 2005).

The potential CTL target epitopes of KIF20A were listed among the HLA-A2 (A*02:01) binders predicted by the BIMAS software and surveyed using HLA-A2 Tgm. All three candidate epitope peptides identified could stimulate the generation of human CTLs that showed KIF20A-specific cytotoxicity in an HLA-A2-restricted manner in all three individuals examined. Importantly, immunisation of the KIF20A-8 peptide, of which the amino-acid sequence is conserved between human and mouse KIF20A, did not induce any signs of autoimmune phenomenon, suggesting the safety of this peptide for using anticancer immunotherapy targeting KIF20A. In this sense, the HLA-A2 is not only a powerful tool for rapid identification of HLA-A2-restricted CTL epitopes but also for evaluating the *in vivo* outcomes of immunotherapy using epitope peptides.

In conclusion, our data showed that KIF20A is a novel TAA and a potential target for anticancer immunotherapy for cancer cells expressing KIF20A at least in an HLA-A2-restricted situation. As KIF20A is highly expressed in a wider range of human malignancies, KIF20A is therefore a promising target for peptide-based immunotherapy for the treatment of malignancies, especially pancreatic cancer. Further investigation of the capability for induction of KIF20A-specific CTLs in pancreatic cancer patients thus remains an issue of great importance for clinical application.

## Figures and Tables

**Table 1:** Expression of the *KIF20A* gene or protein in pancreatic cancer tissues

**Patient**	**RT–PCR**	**IHC**
1	−a	NT
2	−a	−
3	−a	−b
4	+^a^	NT
5	+^a^	+^b^
6	+^a^	+^b^
7	+^a^	NT
8	+^a^	NT
9	+^a^	+^b^
10	+^a^	NT
11	NT	+
12	NT	+
13	NT	+
14	NT	−

Abbreviations: IHC=immunohistochemical analysis; −=negative; NT=not tested; +=positive; RT–PCR=reverse transcription–PCR.

a RT–PCR data are shown in Figure 2.

b Immunohistochemical data are shown in Figure 3.

**Table 2:** Expression of the *KIF20A* gene in pancreatic cancer and various malignancies investigated by cDNA microarray analyses^a^

	***N***	**Positive rate^a^ (%)**	**Average of relative expression ratio**
Pancreatic cancer	6/6	100	31 900
Small cell lung cancer	15/15	100	22
Bladder cancer	30/31	97	20 500
Non-small cell lung cancer	20/22	91	25 800
Cholangiocellular carcinoma	7/11	64	3 780
Breast cancer	29/61	44	322
Prostate cancer	11/36	31	4
Renal cell carcinoma	3/11	27	5
Oesophageal cancer	2/13	15	3
Colorectal cancer	2/31	3	2
Gastric cancer	0/4	0	0

Data are obtained from our previous studies (Nakao *et al*, 1995; Uchida *et al*, 2004; Yoshitake *et al*, 2004; Taniuchi *et al*, 2005; Watanabe *et al*, 2005).

a The relative expression ratio was calculated by dividing the value of the expression of *KIF20A* mRNA in cancer cells by that in the normal counterpart, and a relative expression ratio (cancer/normal tissue) of >5 was considered to be positive.
