# Supplementary material for: Identification of HLA-A2-restricted CTL epitopes of a novel tumour-associated antigen, KIF20A, overexpressed in pancreatic cancer
Source: Br J Cancer. 2010 Dec 21;104(2):300–7. doi: 10.1038/sj.bjc.6606052 (PMC3031900; doi:10.1038/sj.bjc.6606052)
Supplement: Supplementary Table 1 [file 6606052x1.doc]

**Supplementary Table 1. Candidate peptides derived from human KIF20A predicted to be bound to HLA-A2 (*A*0201*)**

A2-binding peptide

Subsequence residue listing

Position

HLA-A2 binding score*

KIF20A-36

KIF20A-35

KIF20A-34

KIF20A-33

KIF20A-32

KIF20A-31

KIF20A-30

KIF20A-29

KIF20A-28

KIF20A-27

KIF20A-26

KIF20A-25

KIF20A-24

KIF20A-23

KIF20A-22

KIF20A-21

KIF20A-20

KIF20A-1

KIF20A-3

KIF20A-2

KIF20A-4

KIF20A-5

KIF20A-6

KIF20A-7

KIF20A-8

KIF20A-9

KIF20A-10

KIF20A-11

KIF20A-12

KIF20A-13

KIF20A-14

KIF20A-15

KIF20A-16

KIF20A-17

KIF20A-18

KIF20A-19

**9-mer**

KVYLRVRPLL

LQAPKDSFAL

QLVHAPPMQL

NLVPFRDSKL

PLLSNEVIWL

SIFSIRILHL

KLNILKESLT

GQASFFNLTV

AQPDTAPLPV

VLQAPKDSFA

LLLKERQEKL

KISELSLCDL

GLLSDDDVVV

LLSDCSVVST

RLLRTELQKL

TLAELQNNMV

LLQEARQQSV

LLSNEVIWL

KMLEPPPSA

LLSDDDVVV

KLGESLQSA

NLLSDCSVV

QLQEVKAKL

KLQQCKAEL

CIAEQYHTV

GLLSDDDVV

TLGRCIAAL

ILPRSLALI

SLALIFNSL

KLNILKESL

ILIKQDQTL

GLQEEELST

TLHVAKFSA

SMYGKEELL

TLAELQNNM

VIWLDSKQI

21.0

21.4

21.4

21.4

22.0

25.0

26.1

26.8

29.0

46.5

65.8

97.0

105.5

119.5

181.8

285.2

484.8

459.4

190.5

198.8

164.0

105.5

87.6

74.8

58.8

51.7

49.1

40.8

40.6

36.6

36.3

30.6

28.8

24.2

20.4

20.2

**10-mer**

66-75

98-107

506-515

455-464

203-212

382-391

625-634

132-141

284-293

97-106

573-582

400-409

11-20

39-48

742-731

788-797

654-663

204-212

715-713

12-20

750-758

38-46

688-696

695-793

809-817

11-19

436-444

179-187

183-191

625-633

781-789

231-239

494-502

556-564

788-796

209-217

†Binding scores were estimated by using BIMAS software (http://www-bimas.cit.nih.gov/).

Note. One peptide which could not be synthesized due to the hydrophobicity of the amino acid sequences was excluded.
